# Orthopaedic Surgery Residency Match After an Early-Exposure Research Program for Medical Students

**DOI:** 10.5435/JAAOSGlobal-D-21-00113

**Published:** 2021-09-10

**Authors:** Emma T. Smolev, Francesca R. Coxe, Sravisht Iyer, Anne M. Kelly, Joseph T. Nguyen, Duretti T. Fufa

**Affiliations:** From the Washington University in St. Louis, St. Louis, MO (Smolev); the Department of Hand and Upper Extremity Surgery, Hospital for Special Surgery, New York, NY (Dr. Coxe and Dr. Fufa); the Department of Spine Surgery, Hospital for Special Surgery, New York, NY (Dr. Iyer); the Department of Orthopaedic Surgery, Sports Medicine, Hospital for Special Surgery, New York, NY (Dr. Kelly); and the HSS Research Institute, Epidemiology and Biostatistics Core, Hospital for Special Surgery, New York, NY (Mr. Nguyen).

## Abstract

**Introduction:**

The purpose of this study was to determine the proportion of students matching in orthopaedic surgery after a structured, early-exposure mentored research program and what factors were associated with those students compared with participants who matched in other specialties.

**Methods:**

Program data were reviewed from 2007 to 2015. Multivariable binary logistic regression analysis was used to evaluate student and research factors associated with orthopaedic surgery match.

**Results:**

Of 174 students, 117 (67%) matched into surgical residency programs, with 49% (n = 85) matching into orthopaedic surgery. The percentage of women matching into orthopaedic surgery (37%) was less than that of men (53%), which, however, increased over the study period. Students who matched in orthopaedic surgery had greater numbers of publications (3.55 [range 0 to 17] average publications) compared with students who matched in other specialties (1.98 (range 0 to 11) average publications). The average number of publications per student increased from 0.79 (±1.44, range 0 to 10, 40%) preprogram to 1.95 (±2.28, range 0 to 11, 71%) postprogram. Measured factors associated with orthopaedic surgery match were publications with program mentor, postprogram first authorship, and total publications.

**Discussion:**

Approximately half of the participants matched into orthopaedic surgery. Analysis showed that research productivity increased after program participation and was statistically associated with increased likelihood of orthopaedic surgery match.

Early exposure to subspecialties is known to play an important role in medical students' specialty match decision-making and, potentially, offers time to improve students' residency application through activities such as research.^1,2^ Orthopaedic surgery is a competitive subspecialty for residency applicants, with more applicants than positions available.^3,4,5,6,7^ In the 2020 residency match, more than 20% of US senior medical students who applied in orthopaedic surgery did not match.^8^ National Resident Matching Program (NRMP) data indicate that orthopaedic surgery programs fill more than 97% of postgraduate year-1 positions annually.^4^ Among factors defining the application process, including US Medical Licensing Examination (USMLE) steps I and II scores, work and volunteer experiences, alpha omega alpha (AOA) Honor Medical Society status, clerkship grades, and medical school ranking, research productivity is an important factor in successful match.^4,6,9-11^ Beginning with the 2021 match, additional challenges in differentiating between large numbers of applications from highly qualified students arose amidst the COVID-19 crisis. More schools went to pass or fail only grades for clinical clerkships, fewer schools designated AOA status, and beginning in the 2023 match, many applicants will have only a pass or fail distinction for U.S. Medical Licensing Examination (USMLE) Step I.

Just behind academic accomplishments including clerkship grades and USMLE scores, orthopaedic surgery program directors rank published research as of great importance.^12^ This importance may grow in the face of changes in grading of clerkships and USMLE mentioned earlier. Literature has shown that early research exposure in medical school increases research productivity, postgraduate research involvement, and, potentially, numbers of physician-scientists.^7,13,14^ These findings already provided a powerful incentive for medical students interested in orthopaedic surgery to perform research before applying to residency. A study analyzing the degree of variability in rotation exposure in orthopaedic surgery residency programs found 63.2% of programs have dedicated research blocks.^15^ As such, exposure to orthopaedic research during medical school may have additional benefit to students' skills as an orthopaedic resident. The fact that almost two-thirds of residency programs have dedicated research rotations signals the increasing emphasis on research skill development, in addition to clinical skills, for orthopaedic surgeons in training.^15^

Since 2007, our institution has offered an 8-week structured, mentored orthopaedic research program (SORP) for students between first (MS1) and second (MS2) years of medical school and with interest in orthopaedic surgery. It provides students with formal faculty research mentorship and offers structured early-exposure to orthopaedic surgery through clinical and surgical shadowing. Studies on similar early-exposure research programs in other specialties suggest they affect later specialty match. Specifically, 52.2% of participants from a Johns Hopkins cardiac surgery 8-week structured research program matched into general surgery or a surgical subspecialty.^16^ The American Pediatric Society 8- to 12-week pediatric structured research program resulted in 36% of participants pursuing pediatrics.^17^ Similarly, the Foundation for Anesthesia Education and Research 8-week structured research program found 58% of participants matched into anesthesiology.^14^

To date, studies examining the effect of structured research programs within orthopaedics are limited. Thus, we investigated the proportion of SORP participants who went on to orthopaedic surgery residency match and what student and research factors were associated with SORP participants who matched into orthopedics compared with those who matched in to other specialties.

## Methods

This retrospective observational cohort study used prospectively collected data from 2007 to 2015. This study range was selected to allow each cohort to complete medical school and match in the expected 4 to 5 years. Nine cohorts with a total of 180 participants (average of 19 students per year, range 12 to 26) completed the SORP. Inclusion criteria were successful completion of the SORP and available residency match data. A total of 174 students met the inclusion criteria (6 students were excluded due to unavailable residency match data). The study period was broken up into two halves to evaluate trends over time: 2007 to 2010 (n = 79) representing the early study period and 2011 to 2015 (n = 95) representing the late study period.

### Structured Orthopaedic Research Program Description

The SORP is an 8-week summer research program centered on a faculty-mentored research project (preclinical, clinical, or biomechanics) aimed at cultivating medical students' interest in orthopaedic surgery by clinical and surgical exposure while also developing their research skills. Mentors are orthopaedic surgeons or collaborating research scientists at our institution. All students who accepted received $2,000 as funding from our institution for their research project. Students may also receive an additional $2,000 for their research project from their home medical institution if funding is available. In addition to research mentorship, students have opportunities for clinical immersion in outpatient and operating room settings. Students also participate in weekly seminar lecture- series and discussion groups to expand their knowledge of medical research and clinical orthopaedics (Table 1). At the end of the 8 weeks, students present their research findings in a formal seminar.

**Table 1:** Example Seminar Schedule for Students in the SORP

Weekly Seminar Schedule for Students in the SORP
Session 1: Introduction to the Program and Discussion of Student Projects
Session 2: Introduction to Musculoskeletal Radiology
Session 3: Introduction to Epidemiology and Biostatistics and Discussion of Student Projects
Session 4; Minimally Invasive Sports Medicine/Physiatry and Case Presentations by the Students
Session 5: Biomechanics Laboratory Tour (Staff) and Case Presentations by the Students
Session 6: Q & A Session—Meet the Clinician-Surgeon
Session 7: Journal Club and Preparing the Presentation
Session 8: Final Presentations

SORP = Structured Orthopaedic Research Program.

This competitive program selects from the applying medical students who are between their MS1 and MS2 years at an LCME-accredited US medical school and in good academic standing. The SORP application includes contact information, undergraduate grade point average and major, medical school, personal statement, resume, and letter of recommendation. After review by the Academic Training Department and orthopaedic faculty SORP committee, selected students are notified and provided a list of prospective faculty mentors and project descriptions.

### Data Collection

An Institutional Review Board exemption was approved for the completion of this study by the Hospital for Special Surgery Institutional Review Board.

Medical students who participated in the SORP between 2007 and 2015 were identified from the Academic Training Department records. Various student, mentor, and research characteristics were recorded and analyzed for their association with later match in orthopaedic surgery. Student characteristics included, sex, and medical school categorized as private or public. Mentor characteristics included mentor sex and number of research publications at the time of mentorship. Details of the SORP research project were gained through the project title, and research characteristics aimed at determining students' pre-SORP and post-SORP number of publications, whether SORP publication was published, student's authorship position, and later publications were recorded are described further.

Publication searches were performed in July 2019 using Scopus, an abstract and citation database with more than 75 million records.^18^ The first search included the participant's first and last name. The “refine” tool was used to limit publications to within 3 years of SORP completion because participants typically graduate from medical school and match into residency in that timeframe. For publications before SORP participation, any publication before July of the SORP cohort year was included. For publications after SORP participation, any publication until August of 3 years after the SORP year was included. To determine the completion of the SORP faculty-mentored project, project titles and mentors' names were compared with the publications listed on Scopus. Scopus was used as the method for publication searches to ensure data collection uniformity. Data collected from each publication included month and year of publication, authorship position, journal, and institutional affiliation. To assess the total number of mentor publications, searches were performed using the aforementioned protocol, and publications were counted up to December of the year before the mentee's SORP participation. Two investigators independently searched for publications (E.T.S. and F.R.C.), and lists were reviewed for any discrepancies.

Residency match results were searched using several strategies. Match lists were reviewed from the student's medical school for specialty choice, residency roster pages were reviewed after Internet search of the student's name, and LinkedIn (linkedin.com) was searched.^19^ Profiles identified through these searches were verified by confirming the student's medical school and year of graduation. Residency specialty and institution were documented.

### Statistical Analysis

Continuous variables were reported as means and standard deviations. Discrete variables were reported as frequencies and percentages. Independent samples *t*-tests were used to compare continuous variables between students who did versus did not match into orthopaedic surgery residency. The chi-square (or Fisher exact) test was used to compare discrete variables between groups. Multivariable binary logistic regression analysis was used to evaluate student and research factors associated with orthopaedic surgery match. Stepwise modeling technique was used to generate a selective model to identify those variables associated with orthopaedic match with best estimates of precision. Variables that achieved final *P* values of 0.10 or below were retained in the final model. Data were analyzed using SPSS version 23.0 (IBM Corp). *P* values of 0.05 or below reached statistical significance.

## Results

### Characteristics of Participants and Mentors

A total of 174 students from 2007 to 2015 were included in the analysis (6 students were excluded for unavailable residency match data). Most participants were men (n = 133, 76%) and attended private medical schools (n = 129, 74%). Before SORP participation, 70 students (40%) had at least one publication for an average of 0.79 (±1.44, range 0 to 10) publications per student in the entire cohort. Male and female SORP participants’ characteristics were similar at baseline (Table 2). The percentage of female SORP students remained stable between the early and late study period (n = 19, 24% from 2007 to 2010; n = 22, 23% from 2011 to 2015).

**Table 2:** Characteristics of SORP Participants

Characteristic	Men (n = 133)	Women (n = 41)	Total
Proportion of private medical school	n = 102 (77%)	n = 27 (66%)	n = 129 (74%) *P* = 0.16
Proportion of public medical school	n = 31 (23%)	n = 14 (34%)	n = 45 (26%) *P* = 0.17
			χ^2^ (1, N = 174) = 1.9 *P* = 0.17
No. of pre-SORP publications (avg, range)	Average: 0.57 Range: 0-8	Average: 0.80 Range: 0-10	Welch two-sample *t*-test: *P* = 0.44

SORP = Structured Orthopaedic Research Program.

The median number of research publications per mentor was 60 (range 0 to 393). Of 174 total mentor-mentee pairs, 33 (19%) mentors were women.

### Residency Match

**Figure 1:**
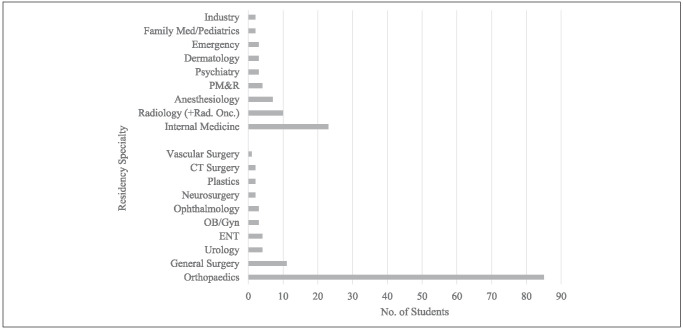
Graph showing residency match results by nonsurgical and surgical specialties for SORP participants (n = 174). The top 9 residency specialties represent nonsurgical specialties, and the bottom 10 residency specialties represent surgical specialties. SORP = Structured Orthopaedic Research Program

A total of 117 students (67%) went on to match into a surgical specialty, with the highest number, 85 (49%), matching into orthopaedic surgery. Other frequent specialty matches included internal medicine (n = 23, 13%), general surgery (n = 11, 6%), radiology (n = 10, 6%), and anesthesiology (n = 7, 4%) (Figure 1). Orthopaedic surgery, not surprisingly, was chosen three times more often than the next most frequently chosen specialty. The percentage of participants who went on to orthopaedic surgery match increased from 46% to 52% throughout the study period.

Of the 41 women, 37% matched in orthopaedic surgery compared with 53% of men. The number of women who matched into orthopaedic surgery increased from 26% to 46% throughout the study period (Table 3). Same sex mentor-mentee pairing did not seem to affect student's match in orthopaedic surgery with 22% of female participants with female mentor matching into orthopaedic surgery compared with 41% of female students with a male mentor.

**Table 3:** Trends in SORP Female Student and Mentors

Variable	Entire Study Period (%)	2007-2010 (%)	2011-2015 (%)
% Female participants	24	24	23
% Female mentors	19	24	15
% Women matched in orthopaedic surgery	37	26	46

SORP = Structured Orthopaedic Research Program.

### Research Characteristics

The average number of publications per student increased from 0.79 pre-SORP to 1.95 (±2.28, range 0 to 11) post-SORP; 32% of students published their SORP project, of which 27% were first author. SORP projects were published at an average of 26 (±9.72) months post-SORP.

In the three years post-SORP, 124 students (71%) published a total of 340 publications, with an average of 2.74 (±2.26) publications per student. Sixty-three students (36%) had first author publications. The proportion of students in the whole cohort with no publications decreased from 60% to 21%. Of the 340 total publications, 49% (n = 165) were published through an outside affiliation, 37% (n = 124) were published with the SORP research mentor, and 15% (n = 51) were published at our institution but with a different faculty member.

SORP students who matched into orthopaedic residency (n = 85) had an average of 3.55 (n = 302) publications per student, which was markedly greater compared with 1.98 (n = 176) for students who did not match into an orthopaedic residency (n = 89) (*P* = 0.001). Further publication detail breakdown when comparing SORP participants who did and did not match into orthopaedic residency can be found in Table 4.

**Table 4:** Publications When Comparing Orthopaedic and Nonorthopaedic SORP Participants

Variable	Totals		Average	
	Orthopaedic Match (n = 85)	Not Orthopaedic Match (n = 89)	Orthopaedic Match (n = 85)	Not Orthopaedic Match (n = 89)
Total First authorship	67	32	0.79	0.36
Total publications	302	176	3.55	1.98
Total publications, pre-SORP	82	56	0.96	0.63
Total publications, post-SORP	220	120	2.59	1.35
SORP project published	30	25	0.35	0.28

SORP = Structured Orthopaedic Research Program.

### Factors Associated With Orthopaedic Surgery Match

Compared with students who matched in other specialties, students who matched in orthopaedic surgery had significantly greater numbers of overall publications (*P* = 0.000), first authorships (*P* = 0.003), and publications affiliated with our institution (*P* = 0.001) (Table 5). No student or mentor factors were associated with orthopaedic surgery match. Multivariate regression analysis identified three factors associated with matching in orthopaedic surgery: publications with SORP mentor (odds ratio [OR]: 5.35, *P* = 0.00), first authorship post-SORP (OR: 3.01, *P* = 0.028), and additional publications post-SORP (OR: 1.21, *P* = 0.076) (Table 6). Publishing a study separate from the SORP faculty-mentored project increased the likelihood of matching by fivefold. First authorship publication post-SORP also increased the likelihood of matching by threefold. For every publication a student had after SORP participation, the odds of orthopaedic surgery match increased by 21%.

**Table 5:** Factors Associated With Orthopaedic Match When Comparing Orthopaedic and Nonorthopaedic SORP Participants

Variable	Orthopaedic Match (n = 85)		Not Orthopaedic Match (n = 89)		*P* Value
	Frequency	SD	Frequency	SD	
No. of publications pre-SORP	1.0	1.7	0.6	1.2	0.126
No. of publications with SORP mentor	1.0	1.2	0.5	0.9	**0.001** ^*^
Total publications with our iInstitution affiliation but not mentor	0.4	1.2	0.2	0.6	0.086
No. of our institution-affiliated first authorships	0.5	0.9	0.1	0.4	**0.000** ^*^
Number of publications post-SORP	2.6	2.5	1.3	1.9	**0.000** ^*^
Total number of Publications (pre-SORP and post-SORP)	3.5	3.3	2.0	2.4	**0.003** ^*^

* *P* value < 0.05 deemed statistically significant.

SORP = Structured Orthopaedic Research Program.

**Table 6:** Student and Research Factors Associated With Orthopaedic Residency Match

Variable	Odds Ratio	95% Cl		*P* Value
		Lower	Upper	
Publications with SORP mentor	5.35	2.09	13.70	**0.000** ^*^
Post-SORP first authorship publication	3.01	1.13	8.05	**0.028** ^*^
Other publications post-SORP	1.21	0.98	1.50	0.076

SORP = Structured Orthopaedic Research Program.

## Discussion

In a time in which traditional metrics used in counseling and mentorship of students interested in orthopaedic surgery are changing, additional data illuminating factors associated with matching in orthopaedic surgery are of increasing utility. The purpose of this study was to determine the proportion of SORP students who go on to orthopaedic surgery match and what measurable factors were associated with those students. We found that 49% of SORP participants went on to orthopaedic surgery match and that research productivity metrics were most strongly associated with orthopaedic match. As expected, research productivity increased after the program across the cohort, with the number of students with at least one publication increasing from 40% pre-SORP to 71% post-SORP. Important research metrics included publications with SORP mentor, post-SORP first authorship, and number of publications post-SORP. However, our study did not account for other known important factors affecting students' decisions and success in pursuing orthopaedic surgery including class rank, USMLE scores, AOA status, number of interviews obtained, interest level, mentorship, or family members in the specialty. Although these factors have undoubtedly traditionally contributed to ultimate match in orthopaedic surgery, they were all determined after the student's SORP participation and are, therefore, out of the scope of this study. Furthermore, in light of the changes occurring in medical education (fewer objective class rankings, less medical schools assigning AOA distinction, and upcoming change of USMLE step I to pass or fail), research and mentorship may play increasingly important roles in student's success in the orthopaedic surgery match in the future.

That roughly half of SORP participants went on to orthopaedic surgery match is consistent with available literature from other specialties, where matched rates ranged from 36% to 58%.^14,16,17^ Similar to the programs previously cited, our SORP is also offered to junior medical students. As a result, many unmeasured factors may have influenced the decision to apply to orthopaedic surgery residency, with some students changing specialty interest during the remaining years of medical school as expected.

For comparison with an orthopaedic-specific program, Nth Dimensions offers a 4-year longitudinal pipeline curriculum focused on mentoring women and underrepresented minority medical students interested in orthopaedic surgery.^20^ They found, not surprisingly, that program completion was associated with increased odds of orthopaedic surgery residency applications.^20^ Because Nth Dimensions is longitudinal in nature, they can more readily associate program completion with orthopaedic surgery match. By contrast, our SORP is a discrete summer program, so more limited comparisons can be made.

Our study revealed that the average number of publications per students more than doubled over the study period for SORP participants from 0.79 to 1.95. This is higher than the national average for the 2013 to 2014 orthopaedic intern class (1.28 [±0.15]).^21^ This is also higher than a comparable program in physiatry (0.8 [±2.8]).^22^ Furthermore, our SORP publication rate of 71% is higher than similar medical student programs, with publication rates from 30% to 50%.^14,16^ In addition, the growth rate in publications was higher in SORP students who matched into orthopaedic surgery (0.96, n = 82 pre-SORP; 2.59, n = 220 post-SORP) compared with SORP students who did not match into orthopaedic surgery (0.63, n = 56 pre-SORP; 1.35, n = 120 post-SORP) (Table 4). In the 2020 NRMP data, matched US orthopaedic applicants had a mean of 14.3 research products (abstracts, presentations, and publications). Of the next most frequent specialty matches from our SORP cohort, internal medicine, general surgery, radiology, and anesthesiology, matched US applicants had a mean of 6.2, 7.1, 6.4, and 5.2 research products, respectively.^8^

Our study is not the first to find that research productivity is associated with successful orthopaedic surgery residency match. Our results showing a relationship between research experience and matching into orthopaedic surgery has been previously demonstrated in the literature.^4,6,9-11^ Among our measured factors, research productivity was found to be most strongly associated with going on to orthopaedic surgery match. Specifically, publishing a study separate from the SORP faculty-mentored project increased the likelihood of matching by fivefold. First authorship publication post-SORP also increased the likelihood of matching by threefold. Schrock et al^6^ found that research experiences and products are higher for residency applicants who match into orthopaedic surgery. In the 2014 NRMP data, matched US orthopaedic applicants had a mean of 6.7 abstracts, presentations, and publications compared with unmatched applicants mean of 3.9.^12^ Our study confirmed this trend; however, our outcome metrics included only publications (excluding abstracts or presentations), explaining the difference in our reported numbers. Our finding that more than one publication with a mentor at our institution correlated with orthopaedic surgery match may suggest that developing a relationship with a mentor is another important factor. Brook et al^23^ found that 84.2% of respondents to an orthopaedic mentorship survey indicated that having a mentor in medical school influenced students' decision to apply into orthopaedic surgery residency. Furthermore, Farkas et al^24^ performed a systematic review of mentorship programs for US medical students and demonstrated an increased match rate into the specialty, improvement in clerkship grades, increased number of publications and presentations, enhanced residency applications, and an increase in successful match for underrepresented students for programs that provided mentorship to medical students and for students who participated in the programs compared with nonparticipating students. Although we cannot directly measure the effect of developing a mentor relationship through research, it is possible that increased publications may serve as an important proxy to mentorship to further evaluate in future, prospective studies.

Orthopaedic surgery has the lowest proportion of female residents among surgical specialties.^25^Although the numbers of female SORP students in the study was lower than those of men, we were encouraged to find the percent of SORP women who went on to match in orthopaedic surgery increase over the study period from 24% to 46%. We did not find that female student-female mentor pairing led to a higher percent matching in orthopaedic surgery; however, our study was not adequately powered to assess this with statistical significance. In line with our findings, a study on mentoring relationships in orthopaedic surgery found 89.3% of survey respondents indicated their mentor's sex did not ultimately influence their specialty choice.^23^ These data suggest that ongoing investigation related not only how to attract female students to programs such as the SORP but also to maintain their interest in pursuing orthopaedic surgery are needed.

Several limitations to this study should be noted. Based on our methodology, we do not know which students applied into orthopaedic surgery but were unsuccessful and matched into another specialty. Because of the retrospective and observational nature of this study that lacks a control group, we cannot account for students' reasoning in choosing one specialty over another or compare students with and without exposure to the program. For the same reason, we also cannot account for other known important factors affecting students' decisions to pursue orthopaedic surgery or their success in the match, including USMLE scores, class rank, the number of interviews obtained, AOA status, interest level, mentorship, or family members in the specialty. In addition, we do not know if, and how many, SORP participants took a year off for research during medical school. Furthermore, publication data may have been underestimated because Scopus was the sole database queried for publications and students' resumes were not reviewed. Moreover, the three-year time cutoff for publications, while chosen for data collection uniformity and correlation with time to apply for residency, may underestimate the true publication rate of SORP projects. Finally, SORP participation may be limited in its direct impact on each student's research productivity and ultimate decision to pursue orthopaedic surgery because multiple complex and unquantifiable factors contribute. Because of the nature of all statistical analyses, we cannot demonstrate causation but, rather, were able to show a statistical association between increased research productivity over the study period and orthopaedic surgery match.

Despite these inherent limitations, several findings can be used to mentor junior students with interest in orthopaedic surgery. These include that (1) students with more publications with a formalized mentor and whose research contributions result in first authorship are more likely to remain interested and match in orthopaedic surgery, (2) student research projects took on average 2 years to reach publication, and (3) sustained research productivity resulted in 21% increased odds in orthopaedic match per published project. At the same time, the findings of this study should only inform part of comprehensive career advising and not to overemphasize the importance of research production given growing concerns over work-life integration for medical students. Further studies are necessary to investigate other important factors associated with students’ pursuit of and success in the orthopaedic surgery match. Other methods, such as qualitative structured interviews, may also improve understanding of the true influence of programs such as the SORP on orthopaedic surgery specialty selection.

## Conclusion

In conclusion, we found 49% of program participants went on to orthopaedic surgery match. Research productivity over the study period was statistically correlated with increased likelihood of orthopaedic surgery match compared with other specialties. These findings support the importance of programs that provide early medical student exposure to mentored orthopaedic surgery research.
